# 

**Published:** 1881-11

**Authors:** 


HERMAN MYNTER, M. D.

### CONTENTS:

| | PAGE. |
|---|---|
| **ORIGINAL COMMUNICATIONS:** | |
| Report of a Case of Inversion of the Uterus Successfully Reduced after Six Months, with Remarks on Reduction in Chronic Inversion. By James P. White, M. D., | 145 |
| Extracts from a Paper on the Obstetrical Forceps. By Prof. James P. White, M. D., | 156 |
| **CLINICAL REPORTS:** | |
| Report on Last Ovariotomy Performed by Prof. J. P. White. By C. M. Daniels, M. D., | 159 |
| An Observation Concerning Catarrhal Inflammation of the Eustachian Tube. By Dr. Lucien Howe, | 160 |
| Surgical Cases. Reported by Herman Mynter, M. D., | 163 |
| Litholapaxy. Reported by Bernard Bartow, M. D., | 168 |
| **SELECTIONS:** | |
| A New Method of Treating Subcutaneous Nævi. By Carey Coombs, M. D., London, | 171 |
| Refrigerators for Sick Chambers, | 171 |
| Effects of Drugs in Lactation, | 172 |
| Puerperal Convulsions, | 175 |
| The Difficulty in Finding a Ball in the Dead Body, | 176 |
| **CORRESPONDENCE,** | 177 |
| **EDITORIAL:** | |
| Professor White and this Issue of the JOURNAL, | 182 |
| Obituary.—Professor James P. White, | 183 |
| —Dr. Walter Cary, | 190 |
| **REVIEWS,** | 191 |

